# Adhesion of the Long Head of the Biceps Tendon: A Case Series

**DOI:** 10.1016/j.asmr.2020.09.001

**Published:** 2020-12-24

**Authors:** Chih-Hao Chiu, Yu-Ching Lin, Poyu Chen, Alvin Chao-Yu Chen, Yi-Sheng Chan, Kuo-Yao Hsu, Alexandre Lädermann

**Affiliations:** a Department of Orthopedic Surgery, Chang Gung Memorial Hospital, Taoyuan, Taiwan; b Bone and Joint Research Center, Chang Gung Memorial Hospital, Linkou, Taiwan; c Department of Medical Imaging and Intervention, Chang Gung Memorial Hospital, Keelung and Chang Gung University, Taoyuan, Taiwan; d Department of Occupational Therapy and Graduate Institute of Behavioral Sciences, College of Medicine, Chang Gung University, Taoyuan, Taiwan; e Department of Orthopedic Surgery, Chang Gung Memorial Hospital, Linkou, Taiwan; f Division of Orthopedics and Trauma Surgery, La Tour Hospital, Meyrin, Switzerland; g Faculty of Medicine, University of Geneva, Geneva, Switzerland; h Orthopedics and Trauma Service, University Hospitals of Geneva, Geneva, Switzerland

## Abstract

**Purpose:**

To present the clinical and imaging findings and results of treatment in patients with intra-articular long head of the biceps tendon (LHBT) adhesion to the undersurface of the rotator cuff found incidentally during shoulder arthroscopy.

**Methods:**

Patients with intra-articular LHBT adhesion to the undersurface of the rotator cuff found incidentally during arthroscopy were included with a minimal 2-year follow-up. Demographic data, images, and physical examinations were recorded. LHBT release, tenotomy, or tenodesis were performed according to the patient’s age and surgeon’s preference.

**Results:**

Twelve patients were included in the study. All of them presented with chronic anterior shoulder pain and positive Speed and O’Brien tests. The average age was 46.8 ± 17 years (range 20-79 years) and the pain sustained from 6 to 96 (average 25.5 ± 28.6) months. Before the operation, 6 patients had a positive Jobe’s test, 1 had a positive lift-off test, and all had positive O’Brien and Speed tests and tenderness over the LHBT. Three release, 4 tenotomy, and 5 LHBT tenodesis were done in addition to other procedures if needed. All range of motion except external rotation, pain visual analog score, and functional outcome scores showed significant improvement at 6 months after surgery. There were no significant differences in range of motion and functional scores between 6 months and 12 months postoperatively. No difference was found in LHBT scores at 6 and 12 months after the operation. Magnetic resonance imaging revealed thickened coracohumeral ligament overlying the LHBT.

**Conclusions:**

Patients who had intra-articular LHBT adhesion to the undersurface of the rotator cuff and underwent release of the adhesion around LHBT, tenotomy, or tenodesis all had good clinical outcomes. The lesion was observed in 2.2% of all shoulder arthroscopies. Although difficult to diagnose before surgery, surgeons should be aware of this unusual condition in patients with chronic and insidious anterior shoulder pain.

**Level of Evidence:**

Level IV, Therapeutic case series.

The long head of the biceps tendon (LHBT) works as a humeral head depressor and a shoulder stabilizer.^1^ It has been regarded as a pain generator in the shoulder joint.^1^, ^2^, ^3^, ^4^, ^5^ The close anatomical proximity of anterior structures, which include the coracohumeral ligament (CHL), the superior glenohumeral ligament, the pulley system, and the rotator cuffs, makes it difficult to find out the true origin of pain. Furthermore, physical examinations and different modalities of imaging have low diagnostic ability.^6^, ^7^, ^8^ The LHBT intra-articular portion has an average length of 24.9 mm from the origin,^9^ which can easily visualized during arthroscopy.^10^ The distal LHBT portion is commonly pathologic due to LHBT tendinopathy, instability, tear, and entrapment (hourglass biceps).^3^^,^^11^ Only a few case reports have demonstrated a unusual lesion of LHBT confluent with the intra-articular rotator cuff.^12^, ^13^, ^14^

The purpose of this series is to present the clinical and imaging findings and results of treatment in patients with intra-articular LHBT adhesion to the undersurface of the rotator cuff found incidentally during shoulder arthroscopy. We hypothesized that release of the adherent portion of biceps with or without tenodesis would yield satisfactory results.

## Methods

### Patient Enrollment

From May 2013 to July 2017, patients receiving arthroscopy for intra-articular examination, rotator cuff repair, LHBT tenodesis/tenotomy, release of refractory frozen shoulder, soft-tissue repairs for shoulder instability, acromioplasty for internal impingement, debridement for calcific tendonitis, and SLAP repairs were included. Patients with intra-articular LHBT adhesion to the undersurface of the rotator cuff found incidentally during arthroscopy were recorded. Two institutions and 2 surgeons participated in patient collection. The adhesion of the LHB to the rotator cuff was assessed during arthroscopy by the 2 surgeons. This study was approved by the institutional review board of the author’s institutes (201900339B0, ethical committee approval; Geneva ethical board; Switzerland; protocol 12-26).

### Clinical and Radiographic Evaluation

Preoperative demographic data included sex, age, involvement of the dominant arm, time from symptom onset to surgery, previous injection, physiotherapy, diabetes, and smoking. Degrees of passive maximum forward elevation, abduction, and external rotation were measured by a goniometer. Internal rotation was measured by the vertebral spinous process that could be reached with the tip of the patient’s thumb and was converted into contiguously numbered groups: T1-12 to 1-12, L1-5 to 13-17, buttock to 18, and greater tubercle of proximal femur to 19.^15^ All patients had preoperative radiographs and magnetic resonance imaging (MRI) to evaluate the conditions of rotator cuff, labrum, capsules and LHBT. Constant score,^16^ pain visual analog scale (PVAS), subjective shoulder value (SSV),^17^ range of motion (ROM), O’Brien test, Speed tests, and tenderness over the LHBT were investigated before operation as well as physical examinations of rotator cuff. After operation, the same evaluation along with LHBT score proposed by Scheibel el al.^18^ also was measured at 6 and 12 months postoperatively. The LHBT score summarizes 3 sections, “pain/cramps” (maximum 50 points), “cosmetics” (maximum 30 points), and “elbow flexion strength” (maximum 20 points), resulting in a maximum of 100 points in total. Elbow flexion strength measurement was calculated using the formula of the LHBT score, comparing the affected with the nonaffected side, giving a percentage for the affected side in comparison to the contralateral side.^18^

### Surgical Technique

All patients were placed in the beach chair position under general anesthesia and interscalene nerve block. Standard posterior and anterior portals were made. The arm was internally and externally rotating to evaluate for the presence of any LHBT instability or laxity of the soft-tissue pulley.^19^ A probe and, if necessary, a 70° scope were used to assist in the dynamic examination to assess LHBT instability.^10^ Then the arm was brought into forward elevation and rotation to see the possible medial/inferior subluxation of the LHBT. In the setting of LHBT instability, this maneuver will lead to tendon entrapment within the joint. The entrapment was relieved with external rotation of the arm. The superior glenohumeral ligament/CHL complex and the subscapularis were individually probed and inspected for the presence of any lesions. The LHBT was then probed and pulled into the joint under traction, allowing for inspection of the proximal portion of the extra-articular tendon. The rotator cuff was assessed during shoulder motion. The subscapularis was inspected during dynamic internal rotation movement, and the supraspinatus was inspected during external rotation and abduction of the shoulder. LHBT release, tenotomy, or suprapectoral tenodesis was performed according to the patient’s age and surgeon’s preference.

### Postoperative Care

Patients with LHBT tenodesis or associated rotator cuff repair were immobilized in a sling for 6 weeks postoperatively. Others wore a sling for the initial 2 weeks right after the surgery. A rehabilitation program consisted of passive ROM exercises for the first 6 weeks, followed by active assisted ROM exercises from 6 to 12 weeks under the supervision of physical therapists in the hospital or self-stretch at home were prescribed for all patients.

### Statistical Analysis

All statistical analyses were performed with SPSS 21.0 for Windows (SPSS Inc., Armonk, NY). Continuous data were described by means and standard deviations. Analysis of variance followed by Bonferroni multiple comparison tests was used to analyze the difference in pre- and postoperative outcome scores for Constant score, PVAS, SSV, and ROM at different time points. Two-tailed *P* values of less than .05 were considered significant.

## Results

From May 2013 to July 2017, 355 arthroscopic rotator cuff repairs, 40 arthroscopic LHBT tenodesis/tenotomies, 20 arthroscopic releases for refractory frozen shoulder, 47 arthroscopic soft-tissue repairs for shoulder instability, 48 arthroscopic acromioplasties for internal impingement and debridement for calcific tendonitis, and 24 arthroscopic SLAP repairs were enrolled. Among them, 12 (10 female and 2 male) patients were identified with intra-articular LHBT adhesion to the undersurface of the rotator cuff. All of them had chronic anterior shoulder pain and positive Speed and O’Brien tests. The average age was 46.8 ± 18.3 years (range 20-79) and the pain sustained from 6 to 96 (average 25.5 ± 28.6) months. A variety of injuries and identified pathology were noted in these patients (Table 1). All patients had an average of 2.8 ± 2 glenohumeral steroid injections a minimum of 3 months before index surgery (range 1-6) and 17.3 ± 12.9 sessions of physiotherapy (range 1-40). Some patients had other kinds of injections, such as platelet-rich plasma or hyaluronic acid, depending on other doctors’ suggestions. Before operation, 6 patients had a positive Jobe’s test, 1 had a positive lift-off test, and all patients had positive O’Brien, Speed tests, and tenderness over the LHBT. Regarding the treatment for intra-articular bicep long head, 3 patients had LHBT release, 4 had tenotomy, and the other 5 had LHBT tenodesis with suture anchor technique at the superior edge of the intertubercular groove (Table 1).

**Table 1:** Patient Demographics

Patient	Age, Y	Sex	Side	Injury	Injury Duration, mo	Diagnosis	Biceps Management	GH Steroid Injection	PT
1	38	F	R	Traction	6	Refractory frozen shoulder	Arthroscopic suprapectoral tenodesis	6	30
2	57	F	R	Fell	12	Supraspinatus calcification	Tenotomy	3	20
3	64	M	R	Postoperative	36	Supraspinatus tear	Tenotomy	3	20
4	31	F	R	Badminton	36	APLSA and Hill–Sachs lesion	Arthroscopic release	1	10
5	46	F	R	Non-specific	15	Supraspinatus calcification	Arthroscopic release	2	30
6	53	F	R	Non-specific	36	Supraspinatus calcification	Arthroscopic release	4	10
7	47	F	R	Traction	7	Refractory frozen shoulder	Arthroscopic suprapectoral tenodesis	1	5
8	20	F	R	Volleyball	96	Biceps instability	Arthroscopic suprapectoral tenodesis	1	2
9	51	F	R	Fell	36	Refractory frozen shoulder	Arthroscopic suprapectoral tenodesis	5	30
10	53	F	L	Fell	8	Refractory frozen shoulder	Arthroscopic suprapectoral tenodesis	6	40
11	79	F	R	Fell	6	Subscapularis tear	Tenotomy	1	10
12	22	M	R	Instability	12	Post-Latarjet	Tenotomy	1	1

APLSA, anterior labroligamentous periosteal sleeve avulsion; F, female; GH, glenohumeral, L, left; M, male; PT, physiotherapy; R, right.

**Fig 1:**
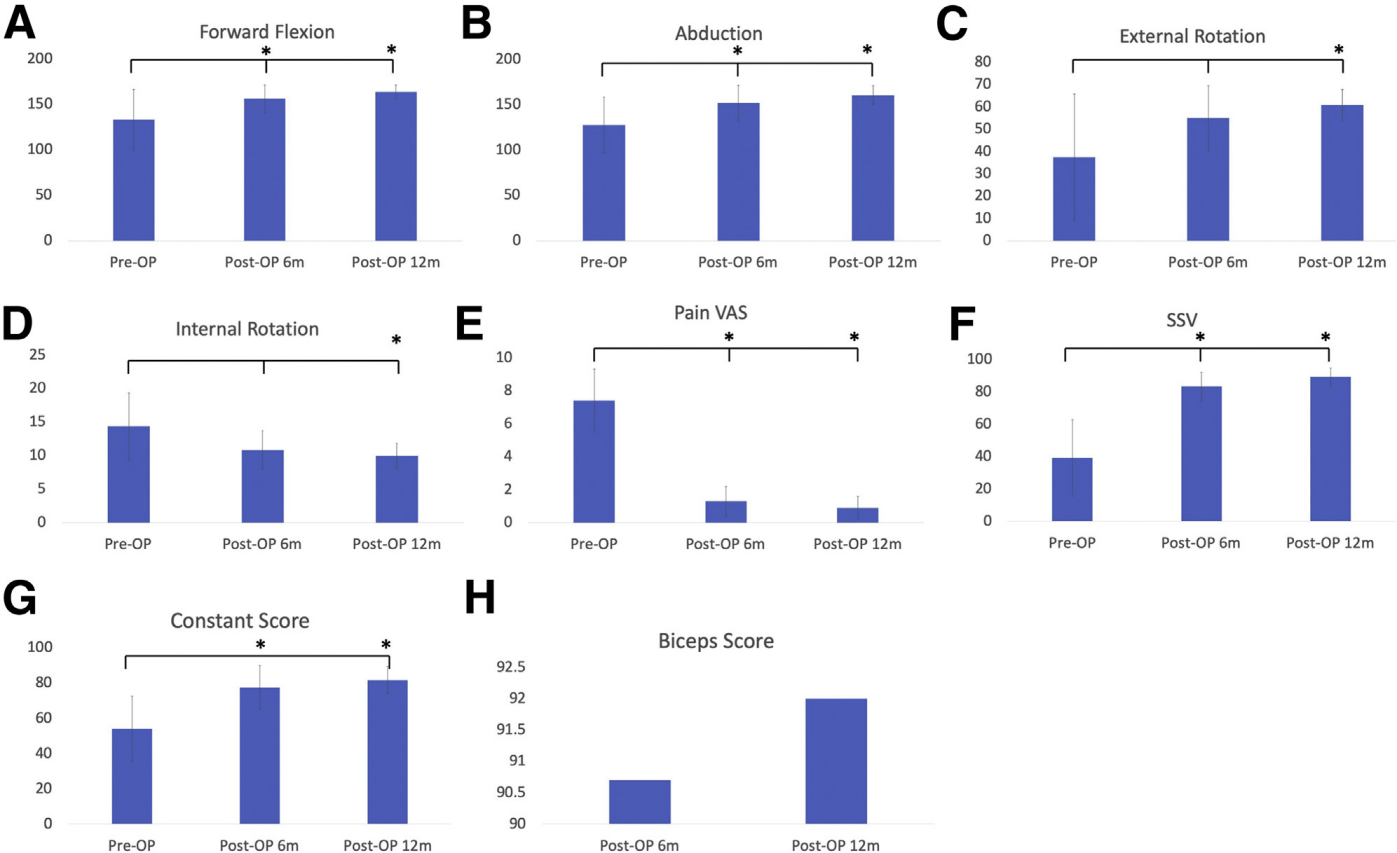
Pre-operative and post-operative ROM and functional score of the treated shoulder. (A) Forward flexion, (B) Abduction, (C) External rotation, (D) Internal rotation, (E) Pain VAS, (F) SSV, (G) Constant score, (H) Biceps score. (VAS, visual analog scale; SSV, subjective shoulder value.)

PVAS, Constant, SSV scores, and all ROM measurements except external and internal rotation of the affected shoulder joints showed significant improvement at 6 months after the surgery (*P* < .001 for all comparisons between pre- and postoperative states). There were no significant differences of ROM and functional scores between 6 months and 12 months postoperatively. No difference was found in LHBT scores at 6 and 12 months after operation (Fig 1, Table 2). Most patients with MRIs in sagittal proton density (PD) fat-saturated images revealed thickened CHL overlying the LHBT.

**Table 2:** Pre- and Postoperative ROM and Functional Outcomes of the Affected Shoulder Joint

	Preoperative	Postoperative 6 Months	Postoperative 12 Months
Forward flexion	133 ± 33.1	155.83 ± 15.1	163.33 ± 7.8
Abduction	127.5 ± 30.4	151.67 ± 19.4	160.00 ± 10.4
External rotation	37.5 ± 28.3	55.0 ± 14.5	60.83 ± 7.9
Internal rotation	14.33 ± 5.0	10.8 ± 2.9	9.92 ± 1.9
Pain VAS	7.42 ± 1.9	1.3 ± 0.9	0.9 ± 0.7
SSV	39.17 ± 23.5	83.3 ± 8.9	89.2 ± 5.6
Constant score	54.0 ± 18.7	77.5 ± 12.4	81.8 ± 7.6
Biceps score		90.7 ± 4.8	92 ± 4.2

ROM, range of motion; SSV, subjective shoulder value; VAS, visual analog scale.

### Case 1

**Fig 2:**
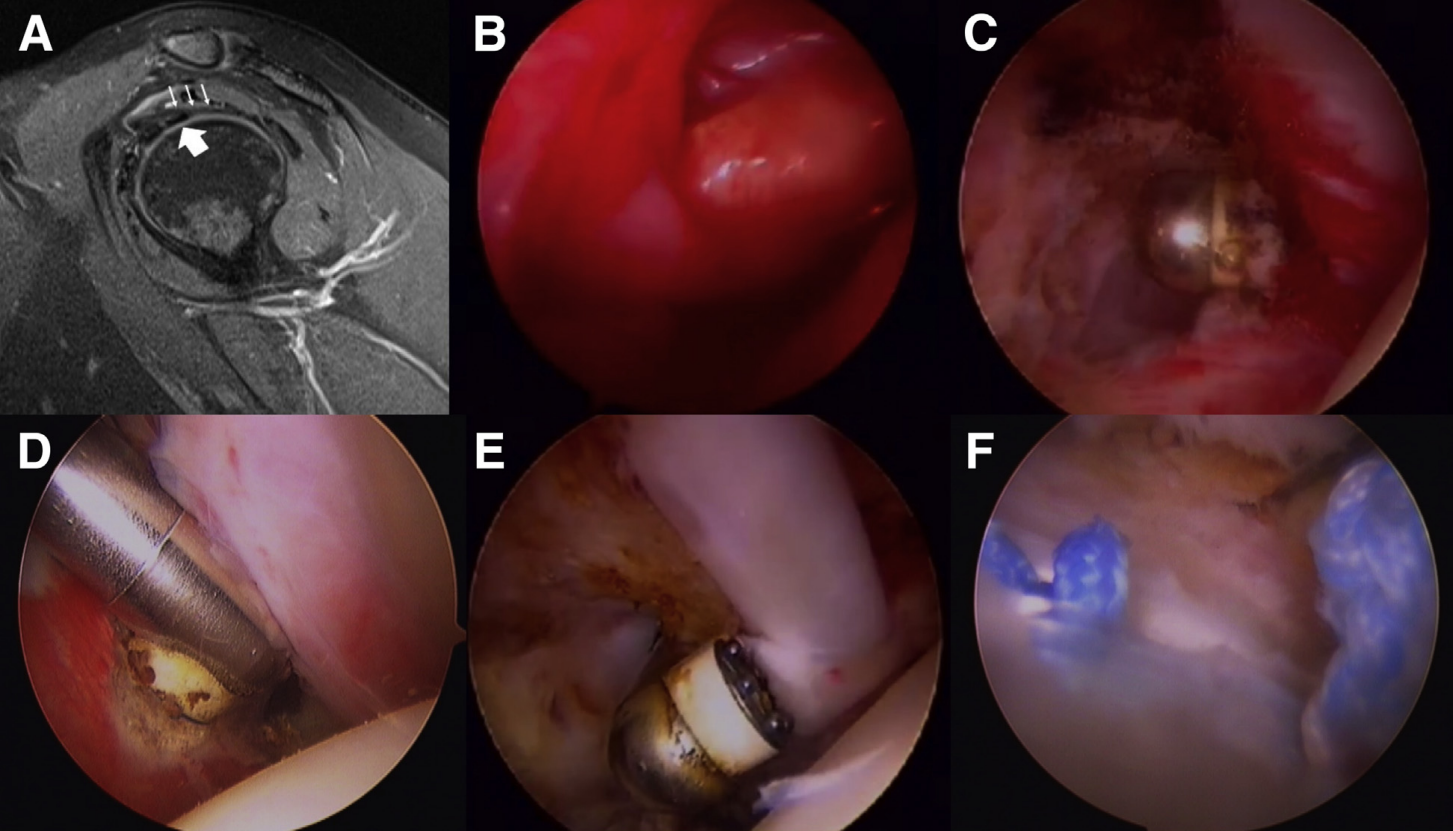
A 38-year-old female patient with frozen shoulder. (A) Sagittal proton density fat-saturated image shows thickened coracohumeral ligament (thin arrows) overlying the biceps tendon (thick arrow). (B) Rotator interval and surrounding capsules were covered by thick and erythematous fibrous tissues accompanying severe synovitis. (C) Release of rotator interval. (D) Release of inferior glenohumeral ligament. (E) Release of intra-articular adhesion of biceps and surrounding tissue. (F) A tenodesis with done with a suture anchor technique.

The patient was a 38-year-old woman working in a convenience store. She was responsible for moving groceries up and down from different shelves. She sprained her dominant right shoulder during work and had pan-shoulder pain and frozen shoulder for 6 months. Conservative treatments were first suggested at another clinic, including 6 courses of glenohumeral steroids, one course of platelet-rich plasma, one course of hyaluronic acid injections, and multiple courses of acupuncture without improvement. The forward elevation, abduction, and external were 80, 80, and 0° and internal rotation to buttock. Sagittal PD fat-saturated image in MRI showed thickened CHL overlying the biceps tendon (Fig 2A). An arthroscopic examination revealed the rotator interval and surrounding capsules were covered by thick and erythematous fibrous tissues accompanying severe synovitis (Fig 2B). After release of rotator interval (Fig 2C) and inferior glenohumeral ligament (Fig 2D), the biceps long head tendon was found adherent to and contiguous with the undersurface of the rotator cuff. After release of intra-articular adhesion of biceps and surrounding tissue (Fig 2E), a tenodesis was performed with a suture anchor technique (Fig 2F).

### Case 2 (With Video Illustration)

**Fig 3:**
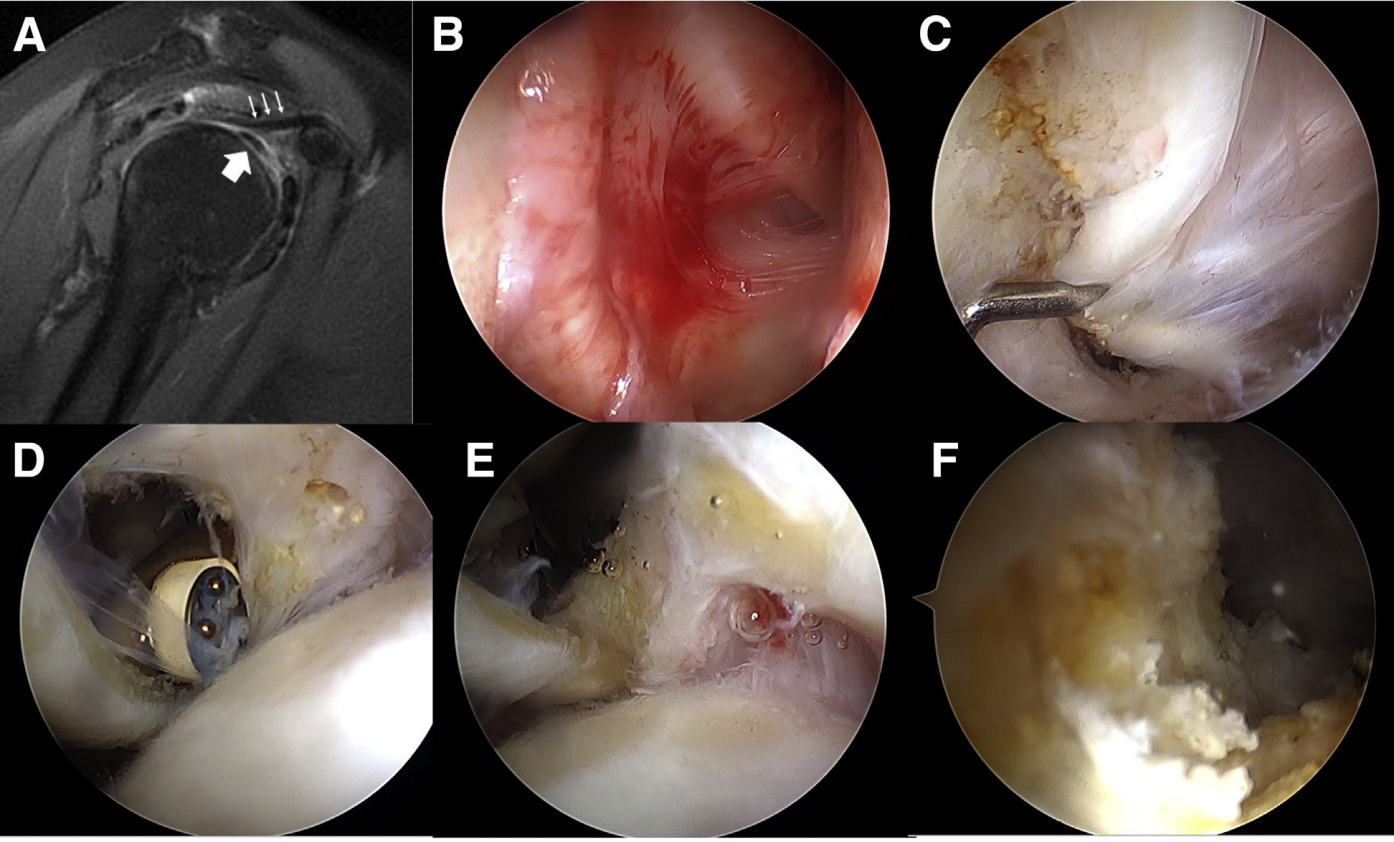
A 57-year-old female patient with insidious shoulder pain and calcific tendonitis in supraspinatus tendon. (A) Sagittal proton density fat-saturated image shows thickened coracohumeral ligament (thin arrows) overlying the biceps tendon (thick arrow). (B, C) Erythematous fibrous tissues covered the bicep anchor around superior labrum and continued alongside biceps tendon into extra-articular section. (D, E) Release of adhesion around biceps. (F) Debridement of calcific spot in the supraspinatus tendon.

This 57-year-old female patient fell with an outstretched right shoulder 1 year ago and presented with an insidious anterior shoulder afterwards. She had poor responses to 3 courses of glenohumeral steroid injection and 20 sessions of physiotherapy within 1 year. Sagittal PD fat-saturated image in MRI showed thickened CHL overlying the biceps tendon (Fig 3A) and a calcific spot in supraspinatus tendon in coronal cut. When arthroscopy was introduced into posterior portal, erythematous fibrous tissues were found covering the bicep anchor around superior labrum (Fig 3B), continuing alongside the biceps tendon into the extra-articular section (Fig 3C). After release of adhesion (Fig 3 D and E), a biceps tenotomy was done along with debridement of calcific spot in the supraspinatus tendon (Fig 3F, Video 1, available at www.arthroscopyjournal.org).

### Case 3

**Fig 4:**
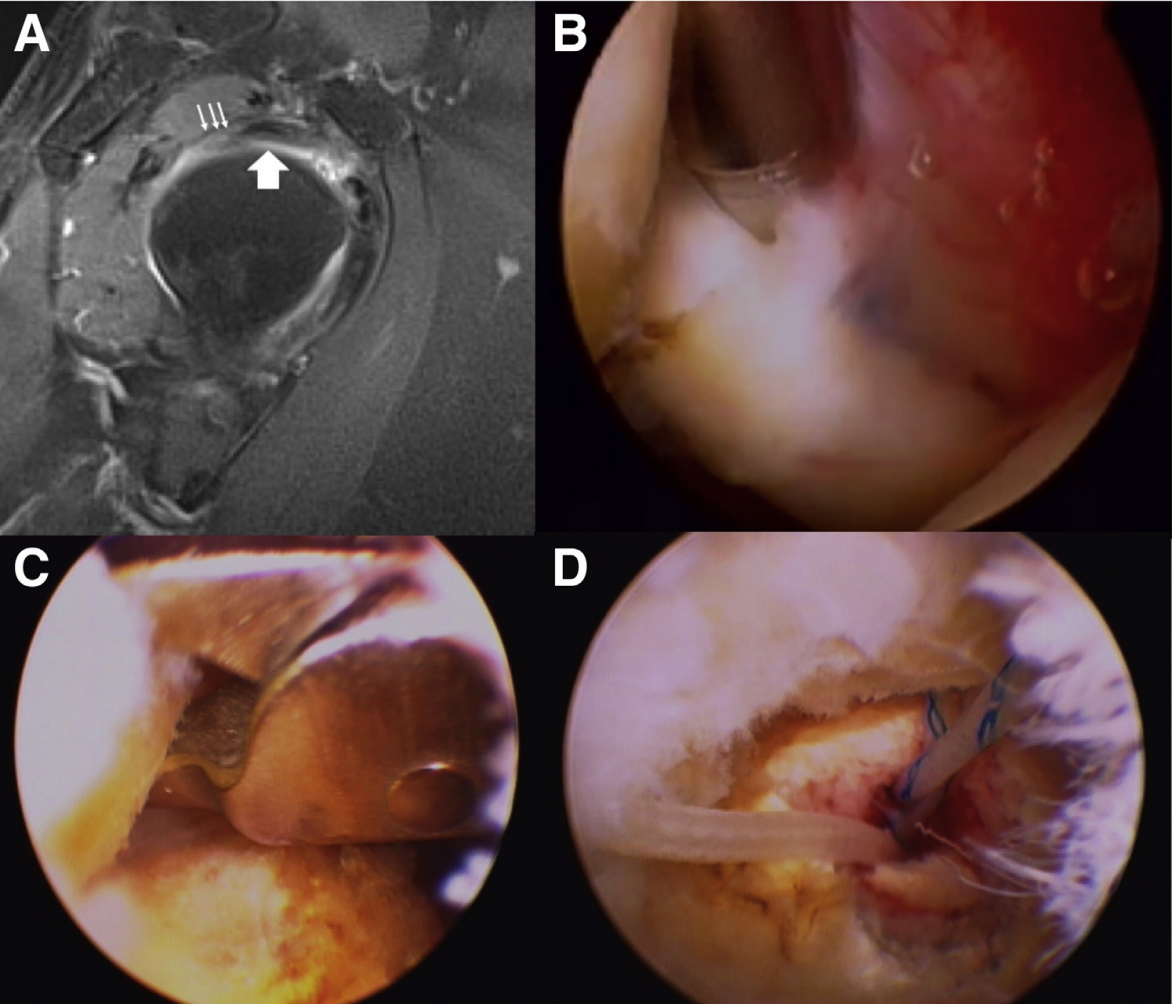
A 64-year-old male patient with 3 previous open shoulder surgeries and supraspinatus tear. (A) Sagittal proton density fat-saturated image shows increasing inflammatory soft tissue (thin arrows) abuts the posterior aspect of biceps tendon (thick arrow). (B) Fibrous tissue was found covering the whole intra-articular part of biceps. (C, D) Supraspinatus repair was done after biceps tenotomy.

A 64-year-old male patient with 3 previous open rotator cuff repairs from the anterolateral approach presented with pain in anterolateral shoulder for 3 years. There was dimpling in the middle deltoid with preserved function. Supraspinatus tear was diagnosed after failure of 3 times of glenohumeral steroid injection and 20 sessions of physiotherapy done at another clinic. Sagittal PD fat-saturated image in MRI showed increasing inflammatory soft tissue abuting the posterior aspect of biceps tendon (Fig 4A). Fibrous tissue was found covering the whole intra-articular part of biceps (Fig 4B). After biceps tenotomy, supraspinatus repair was done (Fig 4 C and D).

### Case 4

**Fig 5:**
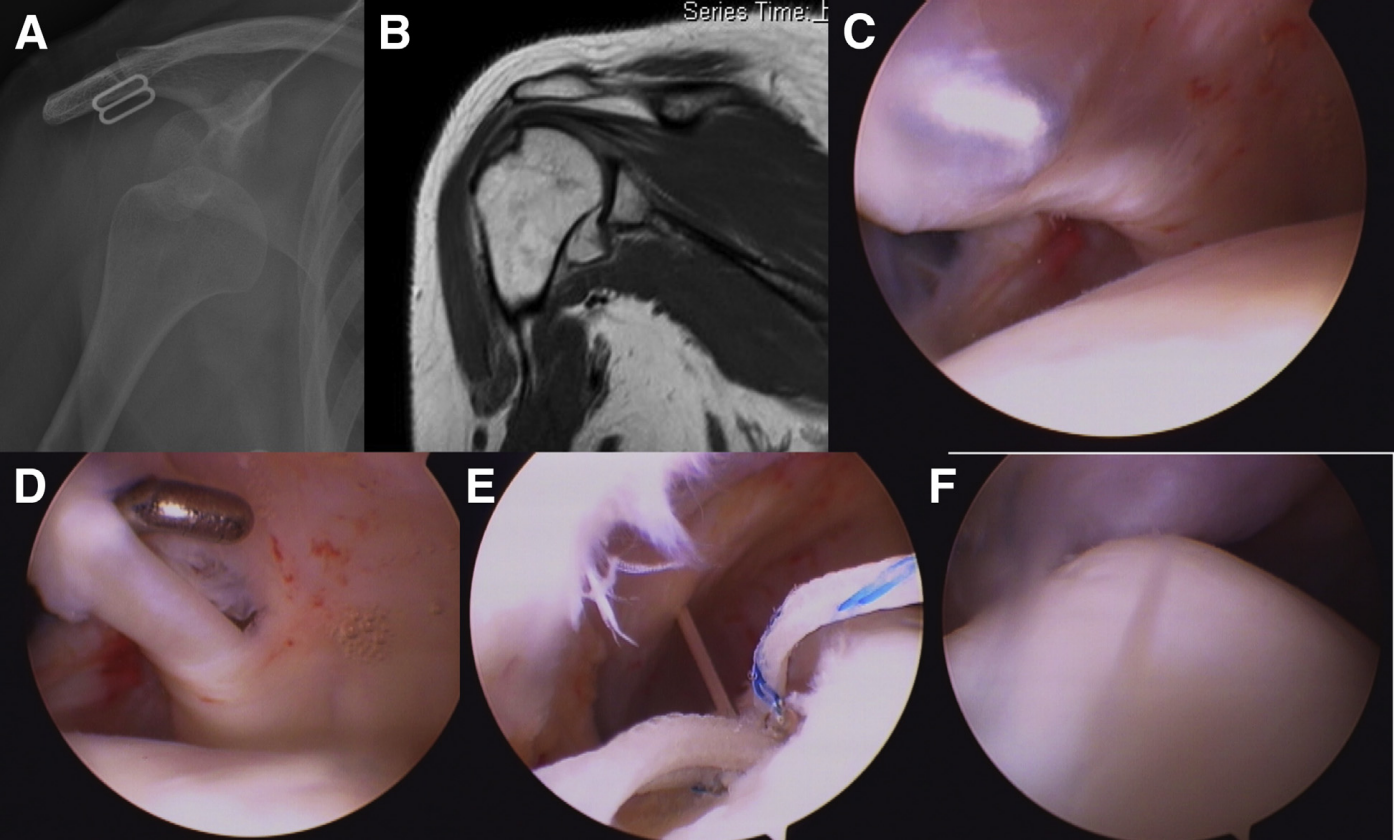
(A, B) A 31-year-old female patient with engaging Hill–Sachs lesion and anterior labroligamentous periosteal sleeve avulsion lesion. (C) Intra-articular biceps long head adhesion to undersurface of rotator cuff was found incidentally before repair (D) Release of the adhesion around biceps. (E, F) Remplissage procedure.

A 31-year-old female patient had more than 10 anterior shoulder dislocations after badminton for 3 years. She presented with anterior shoulder pain occasionally during light overhead sports. Image studies revealed engaging Hill–Sachs lesion and anterior labroligamentous periosteal sleeve avulsion lesion (Fig 5 A and B). Soft-tissue repair and a Remplissage procedure was suggested according to her functional demand and on-tract bone loss preoperatively. Intra-articular biceps long head adhesion to undersurface of rotator cuff was found incidentally before repair (Fig 5C). After release of the adhesion around biceps (Fig 5D), a Remplissage procedure was performed (Fig 5 E and F).

## Discussion

This study reported the clinical image findings and the results of associated treatments in patients with intra-articular LHBT adhesion. Rotator interval’s LHBT extrication is an efficient technique to treat these problems. This finding has several implications relevant to the present condition.

Diagnosis of LHBT adhesion made during arthroscopy should be placed into the differential diagnosis in patients with chronic insidious anterior shoulder pain who do not respond to conservative treatments.^20^ In our series, all of patients had multiple courses of failed injection and physiotherapy with different kinds shoulder pathology leading to index surgery. However, none of them could be easily determined as having intra-articular LHBT adhesion to undersurface of rotator cuff. Thickening of the CHL could only be seen in the sagittal view of MRI, which was very common in frozen shoulder.^21^ There were 3 case reports regarding this unusual lesion with common clinical presentations. In 1998, MacDonald^14^ described a congenital anomaly of the LHBT in arthroscopy whereby the LHBT is adherent to the undersurface of the rotator cuff throughout its course in the shoulder joint. Kim et al.^12^ presented a case with a bursal-side supraspinatus tendon partial tear. They found incidentally an anomalous condition of the LHBT at its attachment to the superior labrum. Throughout its intra-articular course, the tendon was adherent to and contiguous with the undersurface of the rotator cuff. Hammond and Bryant^13^ reported a patient serving in the Navy who presented with symptoms and examinations consistent with a SLAP tear. They planned to perform a SLAP repair and/or capsular release for him. On diagnostic arthroscopy, however, the LHBT was found to be completely incarcerated in tissue and adherent to the rotator cuff from its insertion on the superior labrum to its point of exit from the shoulder joint. In our series, this uncommon lesion was only observed in 2.2% (12/534) of all shoulder arthroscopies. In total, 63.6% (4 refractory frozen shoulder and 3 calcific tendonitis of supraspinatus tendon) of them were shoulder pathologies commonly treated conservatively. For example, many patients with frozen shoulder responded well with conservative treatments such as steroid injections and physiotherapy. However, more than 50% of them still had symptoms and impaired ROM, especially in external rotation at a 7-year follow-up.^22^ In a report by Hagiwara et al.,^23^ approximately 90% of patients with frozen shoulder responded well to intra-articular injections of corticosteroid and physiotherapy, and, accordingly, surgical treatment was required in approximately 10% of the others.

Our findings are relevant to the study of McGahan et al.^24^ Their biomechanical cadaveric study confirmed that intra-articular LHBT adhesion may be similar to in situ tenodesis without proximal tenotomy, which limits shoulder rotation. Kanbe et al.^25^ also reported their series that arthroscopic capsular release for adhesion of the LHBT to the rotator interval improves the sliding movement and thereby shoulder function. In our series, all patients with limited ROM preoperatively had significant improvement after surgery regardless of the LHBT treatments (LHBT release, tenotomy, or tenodesis). The satisfactory result might be due to intra-articular adhesion of LHBT and rotator cuff been cut. Intra-articular LHBT could move freely without adhesion with the surrounding fibrous tissue in LHBT release group. In tenotomy group, LHBT remnant was entrapped by the bicipital groove and transverse humeral ligament in the junction between the joint and bicipital groove. In the tenodesis group, LHBT were fixed with suture anchors at the superior edge of the intertubercular groove.

The connections between our patients may be due to chronic inflammation induced by different pathologies. Three patients had calcific tendonitis in supraspinatus, which is believed to related chronic inflammation.^26^^,^^27^ Four patients with refractory frozen shoulder undergoing arthroscopic release revealed thick fibrous tissues and formation of blood vessels accompanying synovitis around rotator interval. The superomedial capsule and the LHBT were adhered together with synovitis with decreasing sliding motion, which could explain the positive LHBT test before the operation. One patient with type IIB subscapularis tear^28^ and one with small supraspinatus tear also demonstrated severe synovitis in glenohumeral joint. One patient with anterior labroligamentous periosteal sleeve avulsion and Malgaigne (Hill–Sachs) lesion due to badminton complained about anterior shoulder pain along with positive apprehension. Another volleyball player with 8 years of unexplained anterior shoulder pain without dislocation also demonstrated adherent LHBT to undersurface of rotator cuff, which might be due to repetitive overhead sports when serving the volleyball. One patient with previous open Latarjet procedure had insidious anterior shoulder pain after surgery until LHBT adhesion to rotator cuff diagnosed in follow-up arthroscopy.

All patients had significant improvement regarding ROM and functional scores after the surgery. We did not record LHBT scores preoperatively because LHBT lesions were not suspected in all patients before the index surgery. There was no difference in LHBT scores at 6 and 12 months after operation regardless of the different treatments of LHBT. The aforementioned condition implied either surgical treatment for intra-articular LHBT adhesion to undersurface of rotator cuff provide acceptable outcome.

### Limitations

This series has some limitations to be acknowledged. First, the different shoulder pathologies and treatments for LHBT and other coexistent lesions were not controlled. This lesion has only been found in 3 case reports previously and encompassed 2.2% of all shoulder arthroscopies performed by the authors. The main objective of this study is to report the unusual lesion and remind shoulder surgeons to take it into consideration if patients have chronic and insidious anterior shoulder pain. In contrast, the surgical choices for LHBT lesion from release, tenotomy, or tenodesis were based on the patient’s age and surgeon’s preference. The average ages for patients receiving release, tenotomy, and tenodesis were 43.3, 55.5, and 41.8 years. All patients presented with good LHBT scores after surgery. Second, this lesion is not easily to be determined by physical and radiologic examination before surgery. Although the O’Brien test is highly sensitive (97.8% and 95.7%, respectively) and revealed high negative predictive value, Speed’s test is nonspecific but sensitive for macroscopic LHBT/labral pathology.^6^^,^^29^ MRIs of patients in our series only revealed thickened CHL in the sagittal view, which is not easily to differentiate from frozen shoulder. We could only determine this unusual lesion in shoulder arthroscopy. Third, not all patients were presented with a clear traumatic history. Among them, 2 patients with calcific tendonitis of the supraspinatus tendon and chronic pain denied obvious injury before the pain. However, severe synovitis was observed during arthroscopy in all our patients. Chronic inflammation of synovium can cause mechanical and histologic changes in adjacent capsular tissue as well as adhesions within a tenosynovial compartment, which can conceivably result in stenosis of a compartmentalized gliding LHBT tendon.^30^ Hence, uncontrolled chronic inflammation may cause this unusual lesion.

## Conclusions

In this study, patients who had intra-articular LHBT adhesion to the undersurface of the rotator cuff and underwent release of the adhesion around LHBT, tenotomy, or tenodesis all had good clinical outcomes. The lesion was observed in 2.2% of all shoulder arthroscopies. Although difficult to diagnose before surgery, surgeons should be aware of this unusual condition in patients with chronic and insidious anterior shoulder pain.

## Supplementary Data

Video 1 Case 2, a 57-year-old female patient with calcific supraspinatus and biceps adhesion found unexpectedly during arthroscopy, is shown. Erythematous fibrous tissues covered the bicep anchor around superior labrum and continued alongside biceps tendon into extra-articular section. Release of adhesion around biceps was performed.

ICMJE author disclosure forms
